# Supplementary material for: Structure and function of the EA1 surface layer of Bacillus anthracis
Source: Nat Commun. 2023 Nov 3;14:7051. doi: 10.1038/s41467-023-42826-x (PMC10624894; doi:10.1038/s41467-023-42826-x)
Supplement: Supplementary file 1 — Supplementary Information [file 41467_2023_42826_MOESM1_ESM.pdf]

Supplementary information for:

Structure and function of the EA1 surface layer of *Bacillus anthracis*

Adrià Sogues [1,2#], Antonella Fioravanti [1,2], Wim Jonckheere [1,2], Els Pardon [2,3], Jan Steyaert [2,3] & Han Remaut [1,2 #]

1.Structural and Molecular Microbiology, VIB-VUB Center for Structural Biology, VIB, Pleinlaan 2, 1050 Brussels, Belgium
2. Structural Biology Brussels, Vrije Universiteit Brussel, Pleinlaan 2, 1050 Brussels, Belgium
3. Structural Biology Brussels, Vrije Universiteit Brussel, VUB, Brussels, Belgium.
VIB-VUB Center for Structural Biology, VIB, Brussels, Belgium.

#Correspondance: Adria.Sogues.Castrejon@vub.be & Han.Remaut@vub.be

This PDF file includes:

Supplementary Figures 1- 20

Supplementary Tables 1 and 2

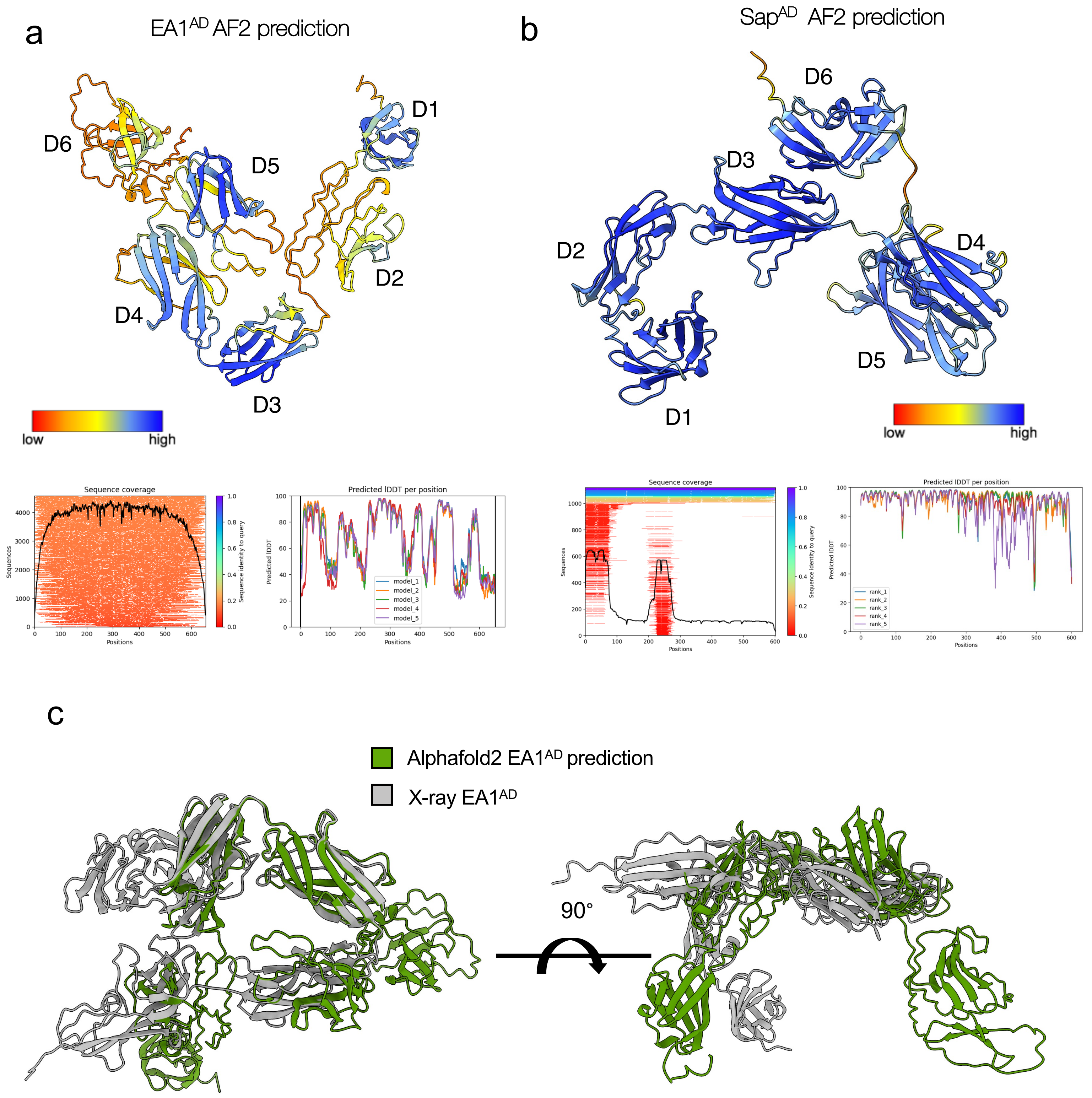

Supplementary Figure 1. Alphafold2 (AF2) prediction of EA1$_{AD}$ and Sap$_{AD}$. a . (top) Cartoon representation of the assembly domain of EA1 predicted by AF2 colored according to the confidence of the prediction (scale on the bottom right). As observed, the prediction presents regions of low confidence, nevertheless, six distinct domains can be distinguished. (bottom) Sequence coverage and predicted LDDT plot per position. DeepMind reports pLDDT > 90 as high accuracy predictions, between 70 and 90 as good backbone predictions, and pLDDT < 70 as low confidence and to be treated with caution. **b**. Alphafold2 prediction for Sap^{AD}, sequence coverage and LDDT plot. **c**. EA1^{AD} superposition of the AlphaFold2 predicted structure (green) and experimentally X-ray determined in this study (grey). Rmsd across all residues is 51.88Å.

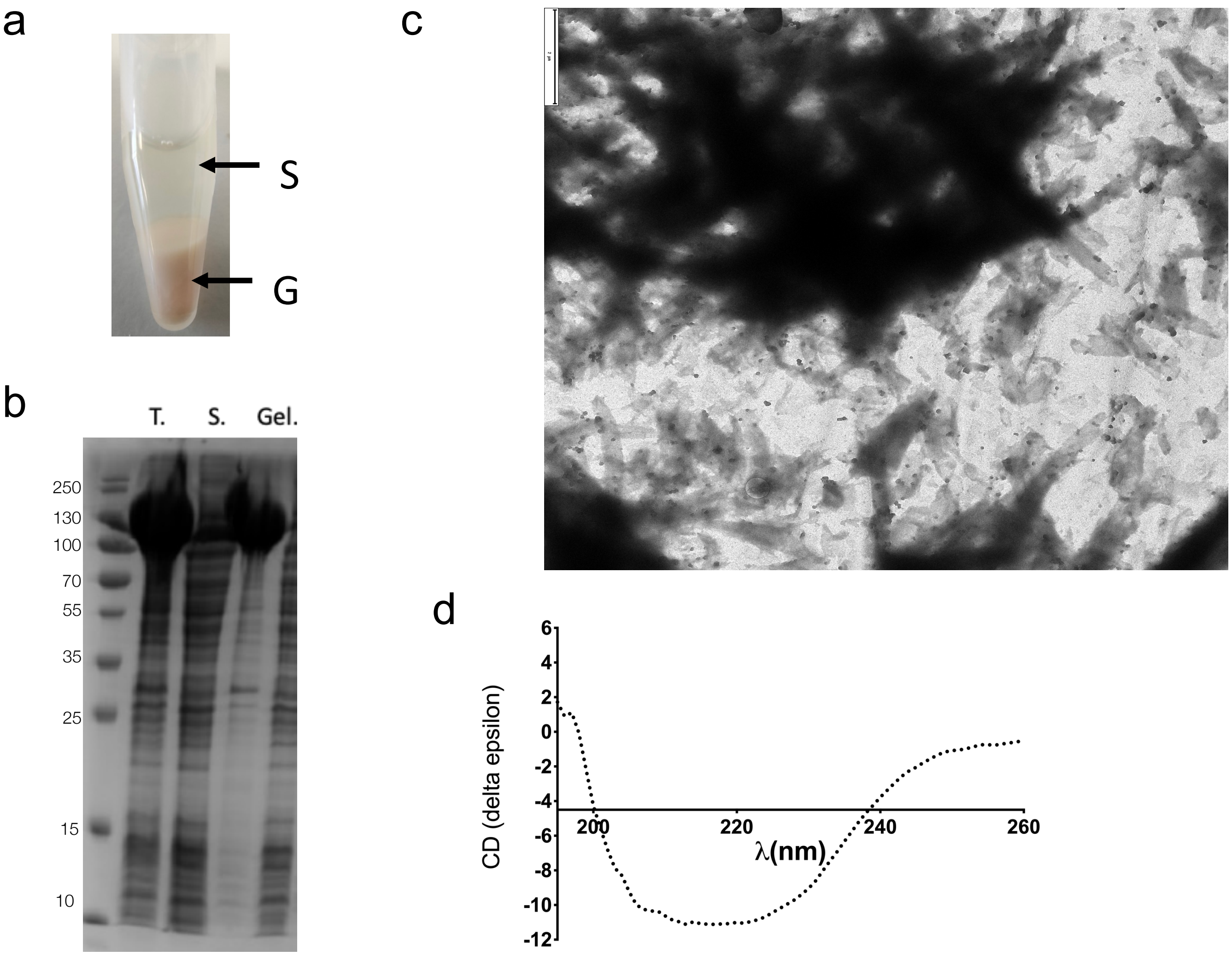

Supplementary figure 2. Purification and refolding of EA1 a. After lysing and centrifuging the culture *E. coli* BL21 expressing EA1 FL a big pellet with a gel-like consistency and a clear supernatant can be observed (G and S respectively as indicated in the figure). **b**. SDS-PAGE of the sample shown in panel a. T- total lysate; S- supernatant; Gel- pellet after centrifugation. Most of the EA1 is found in the pellet after overexpression. **c.** $EA1_{FL}$ was imaged by negative-stain TEM after resuspending the EA1 pellet in PBS. $EA1_{FL}$ forms conglomerates of 2D sheets. The scale bar (top left) is 2 µm. **d**. Circular dichroism spectra of $EA1_{FL}$ after removing the denaturing agent. The minima at 218 nm indicated high content in β sheets. The experiments were repeated independently at least three times with similar results.

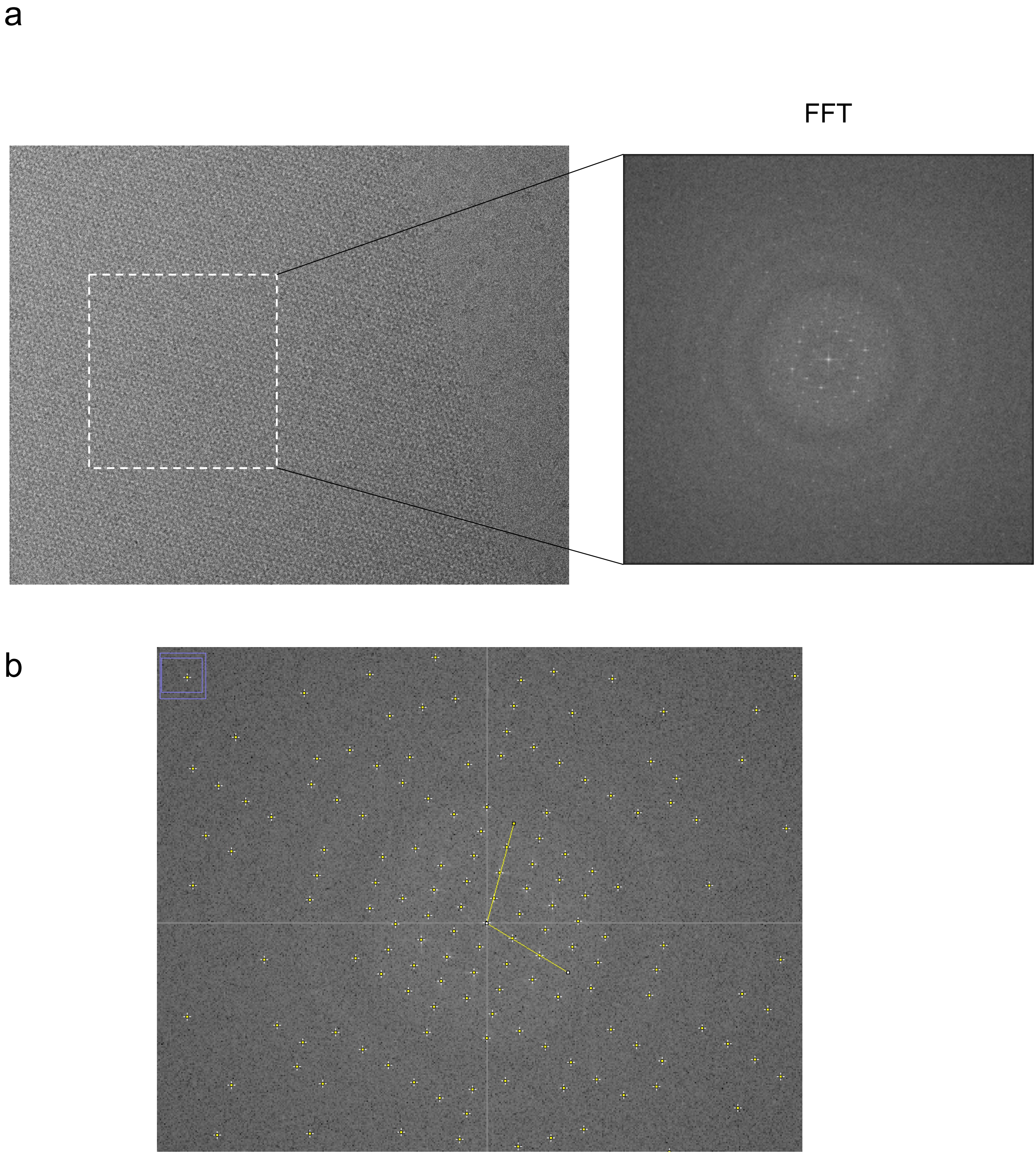

In vitro EA1 cell parameters: α= 73.78 Å, β=87.84 Å, $\boldsymbol{\gamma}$=107°

Supplementary figure 3. Lattice dimensions calculated for in vitro reconstituted S-layers. a. $EA1_{FL}$ monolayers (right) imaged by Cryo-EM. A single monolayer was selected, and calculated power spectra (left) was generated by Fourier transform using ImageJ. **b**. For clarity, Spots were detected (maxima, yellow dots). Calculated $EA1_{FL}$ lattice parameters are α= 73.78 Å, β=87.84 Å, $\boldsymbol{\gamma}$=107°.

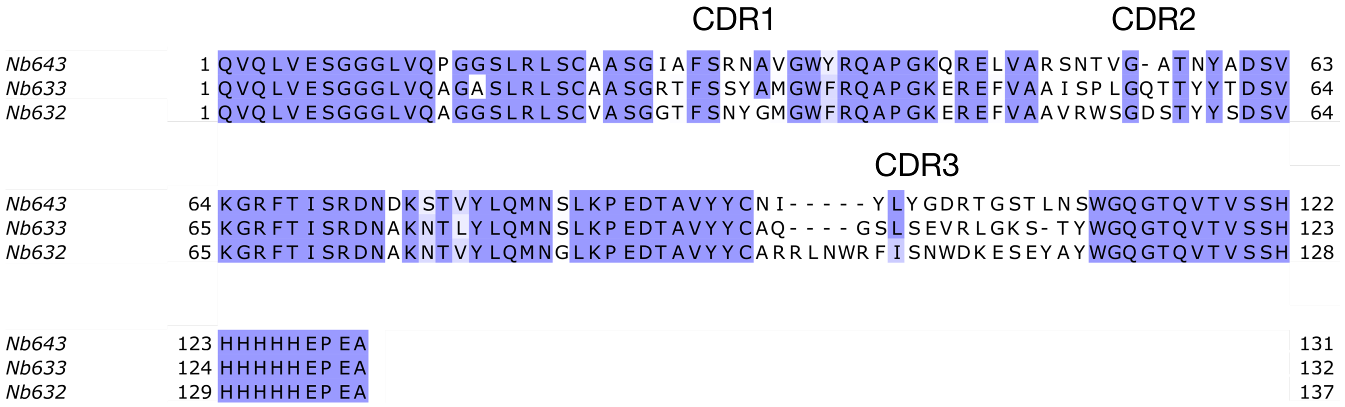

Supplementary Figure 4. Sequence alignment of the three selected EA1-binding nanobodies. Based on the sequence diversity of the different complementarity determining regions (CDR) (in particular CDR2 and CDR3), three nanobody families can be distinguished that would correspond to different binding epitopes.

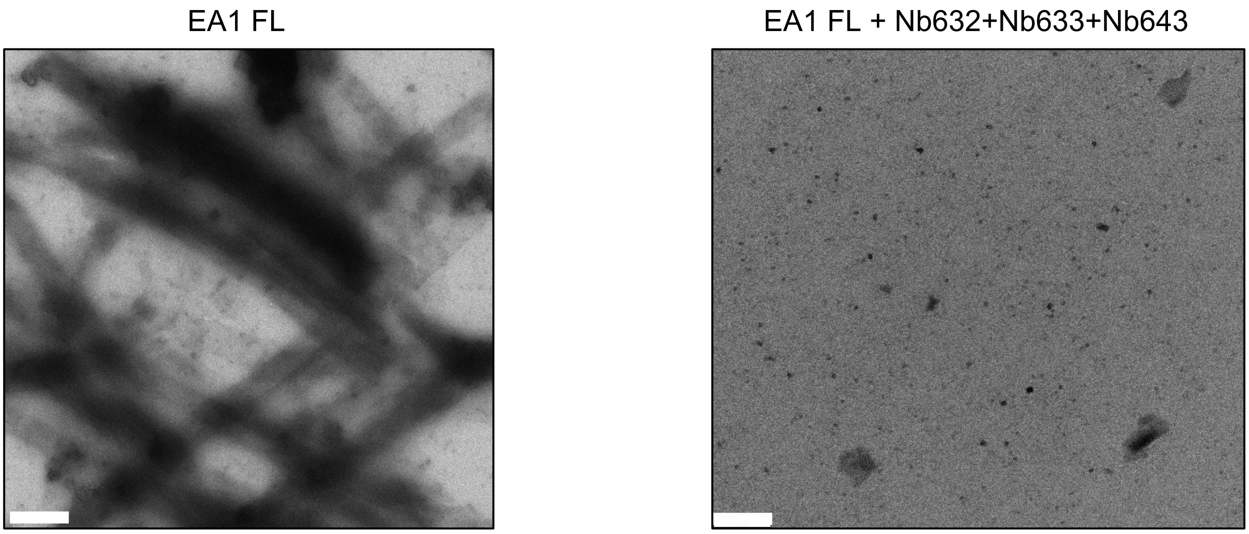

Supplementary figure 5. Effect of depolymerizing nanobodies on EA1 S-layers. a, Representative negative-stain TEM images of $EA1_{FL}$ without (left) or with an equimolar mix of Nb632+633+643 (right) after 60 minutes incubation. Absence of EA1 S-layers were also in the presence of the nanobodies. Scale bar is 500 nm. This experiment was repeated independently at least three times.

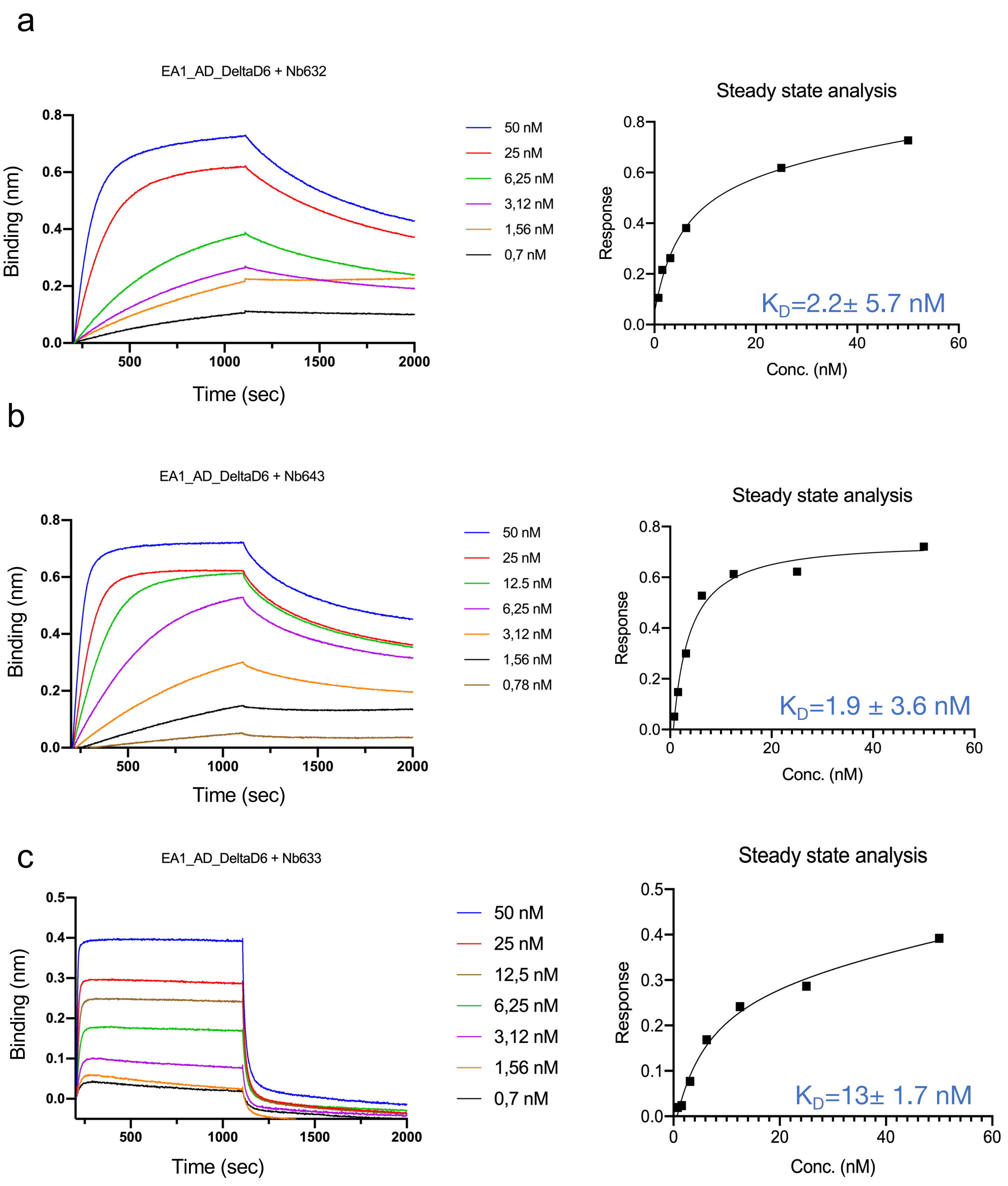

Supplementary figure 6. Biolayer interferometry experiments. a, **b**, **c**, Affinity measurement of Nb632 (top) and Nb643 (middle) and Nb633 (bottom) with EA1$_{\Delta D6}$. We used EA1$_{\Delta D6}$ as it does not polymerize and still presents the epitope for Nb632, Nb633 and Nb643. Left panels indicate representative BLI binding sensorgrams color according to the concentration of nanobody. Right panels are representative of steady state analysis where BLI response is plotted versus concentration. The K_D is indicated in blue within the plot. The experiments were repeated independently at least three times with similar results.

a

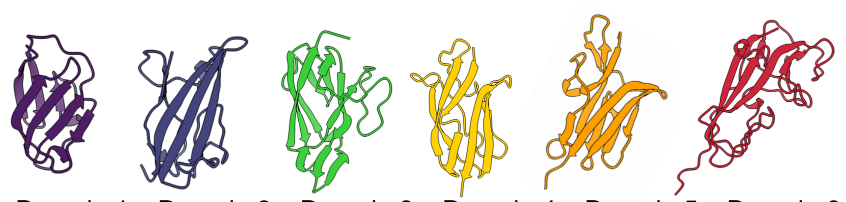

b

RMSD (Å)	D1	D2	D3	D4	D5	D6
D1	0	9.529	14.235	13.893	15.653	13.713
D2		0	14.556	16.093	16.136	14.059
D3			0	17.966	7.435	18.711
D4				0	11.708	20.732
D5					0	16.373
D6						0

c

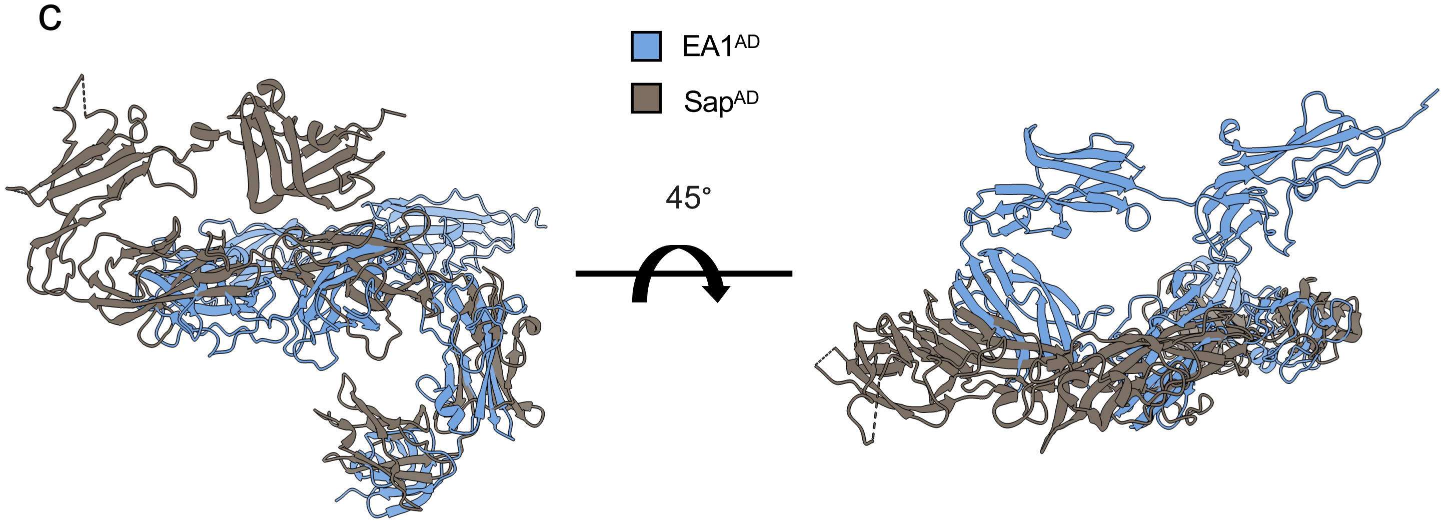

d

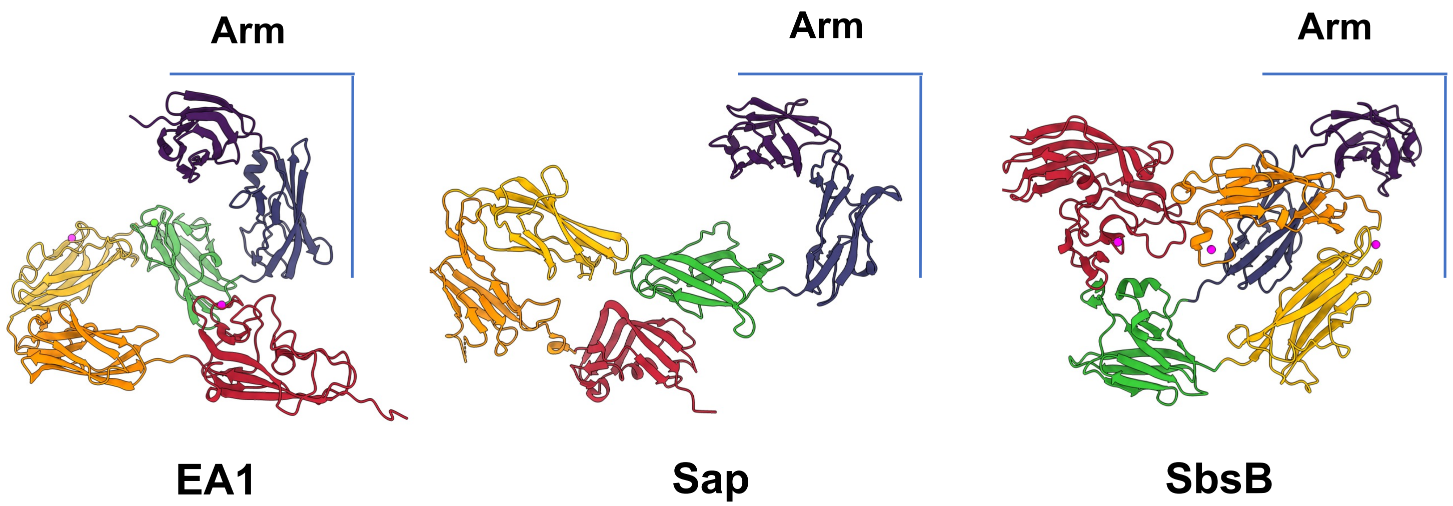

Supplementary figure 7. Domains of EA1 and structural comparisons. a. Ribbon representation of individual domains from D1 to D6 of EA1 in purple, blue, green, yellow, orange and red respectively. **b**. Root mean squared deviation (rmsd) table for structural alignment of EA1 domains perform with ChimeraX. All domains present an overall β-sandwich structure except D6 which is the most structurally distinct subdomain with many loop regions. **c**. Structural superposition of EA1 and Sap AD (rmsd of 35.18 Å). The best structural agreement between the two structures lies in the D1 and D2 region. **d**. Structures of EA1, Sap (6HHU) and SbsB (4AQ1) in ribbon representation. Domains 1 to 6 are identically coloured. Calcium atoms are coloured in magenta. Arm formed by domains 1 and 2 is indicated in each structure.

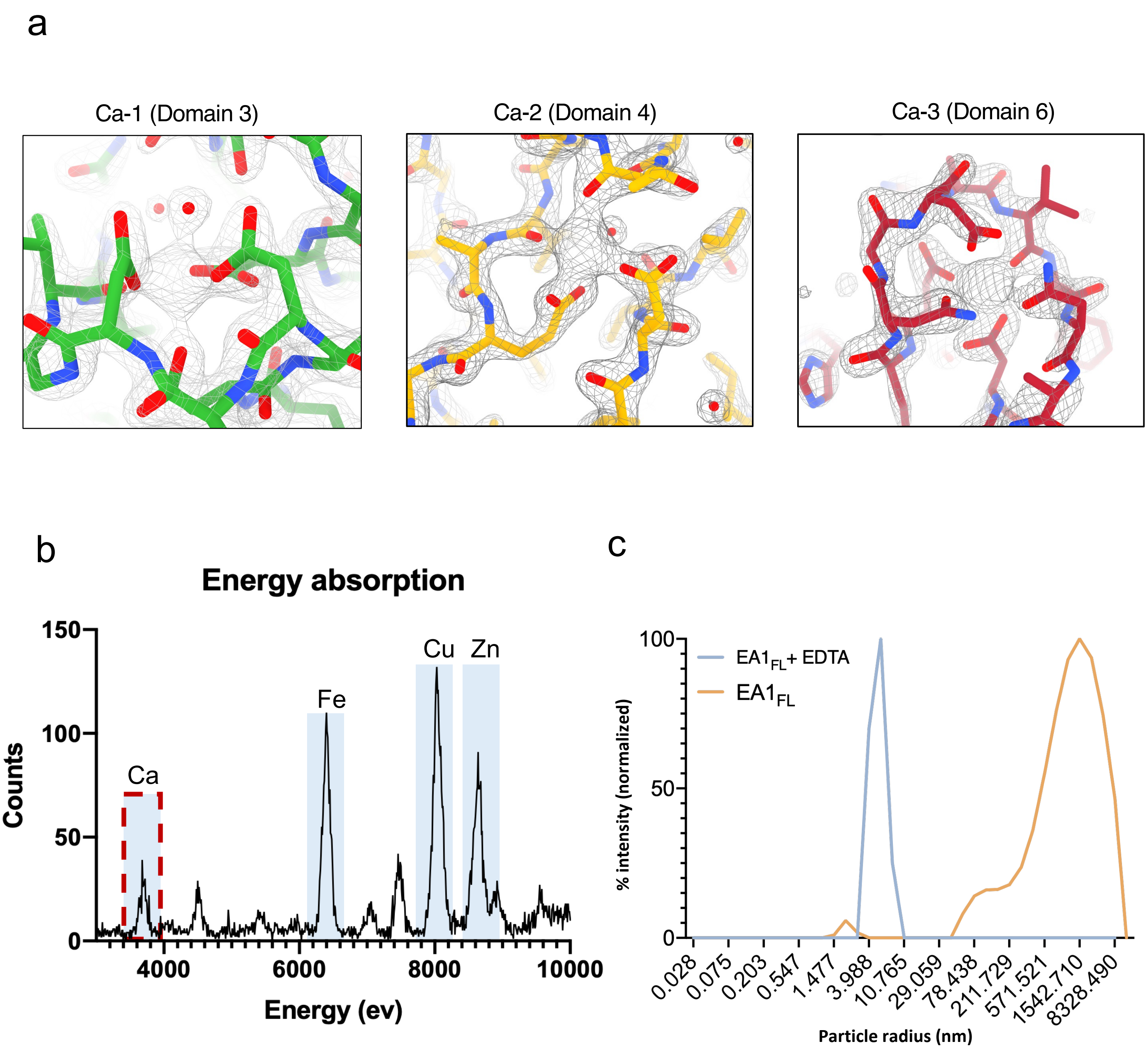

Supplementary figure 8. EA1 binds calcium. **a.** Stick representation of the three different metal binding sites and the final 2Fo-Fc electron density map (contoured at 1.5 σ). **b.** X-ray fluorescence spectrum of the EA1 crystals. Four main peak were detected for Ca, Fe, Cu and Zn, however, data collection above the Fe, Cu or Zn K-edge absorption peaks did not result in an anomalous EA1AD diffraction. Thus, the chemical environment found at the metal position in the structure and the calcium peak observed (the theoretical value of calcium absorption K-edge is 4.0381 KeV) we can conclude that calcium is the metal bound to EA1. **c**. DLS after refolding in presence of 1 mM EDTA (blue) or in absence of EDTA (orange).

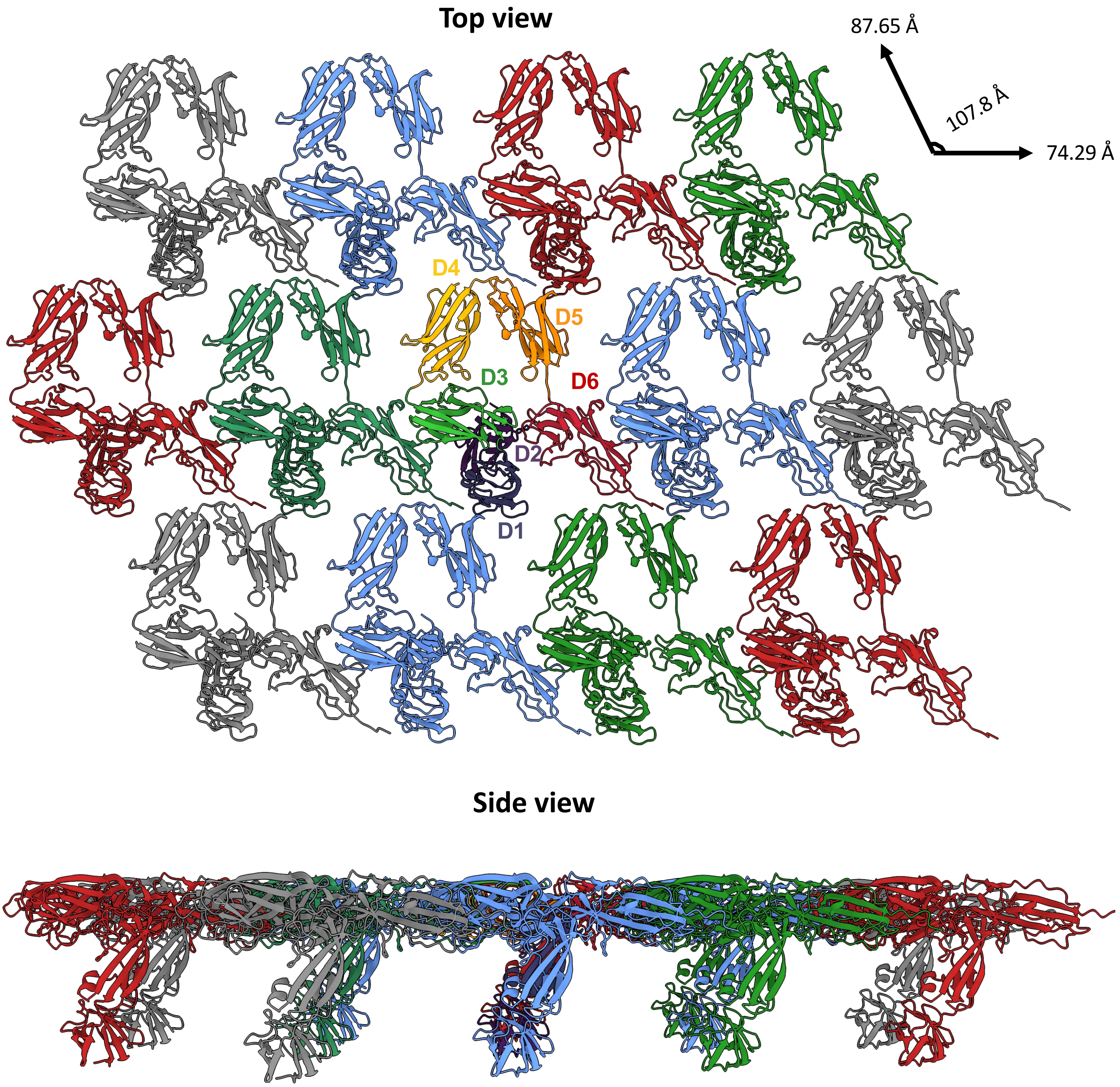

Supplementary figure 9. X-ray lattice contacts of EA1. Thirteen asymmetric units of the EA1 crystal from the sample plane are shown. For clarity, nanobodies are not shown. EA1 forms a continues lattice with domains lying in the sample plane (domain 3 to 6). A crossed-pattern can be identified that involved D3, D6, D4 and D5 of three different EA1 molecules. Pseudo-symmetrical vectors are shown on top of the lattice. Sideview shows how domains 1 and 2 are not part of the lattice due to the nanobodies binding. For clarity, nanobodies are not shown.

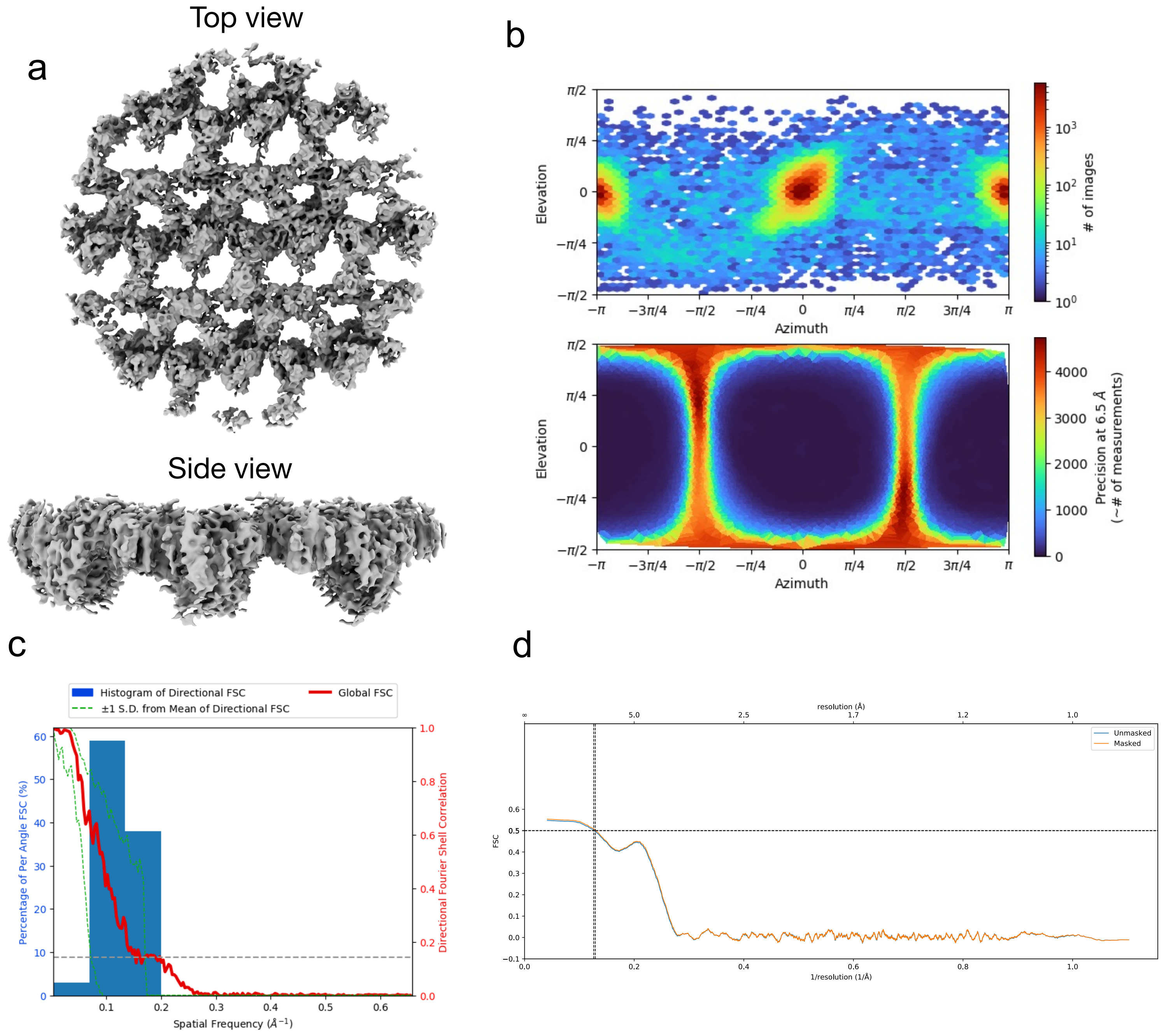

Histogram and directional FSC for EA1
Sphericity = 0.633 of 1. **Global resolution = 6.61 Å**

Supplementary figure 10. Cryo-EM data and processing. **a**, Top and side view of the cryo-EM map obtained from EA1 monolayers. Top view presents a crossed-shaped pattern whereas side the side view is not completely resolved due to missing view. Nevertheless, protrusion pointing down can be inferred that would correspond to the density of domain 1 and/or SLH. **b.** Viewing Direction Distribution (top) and Posterior Precision Directional Distribution (bottom) calculated by CryoSparc after non-uniform refinement. **c**. FSC curves obtained from the 3DFSC server. Plots of the global half-maps FSC (solid red line) together with the spread of directional resolution values defined by ±1σ from the mean (green area encompassed by dotted green lines) and a histogram of 100 such values evenly sampled over the 3D FSC (blue bars, left axis). Global resolution estimated at 6.61. **d**. Map vs model FSC for the D3-D6 fitted into the cryoEM map. The estimated resolution at 0.5 FSC cutoff is 7.7Å

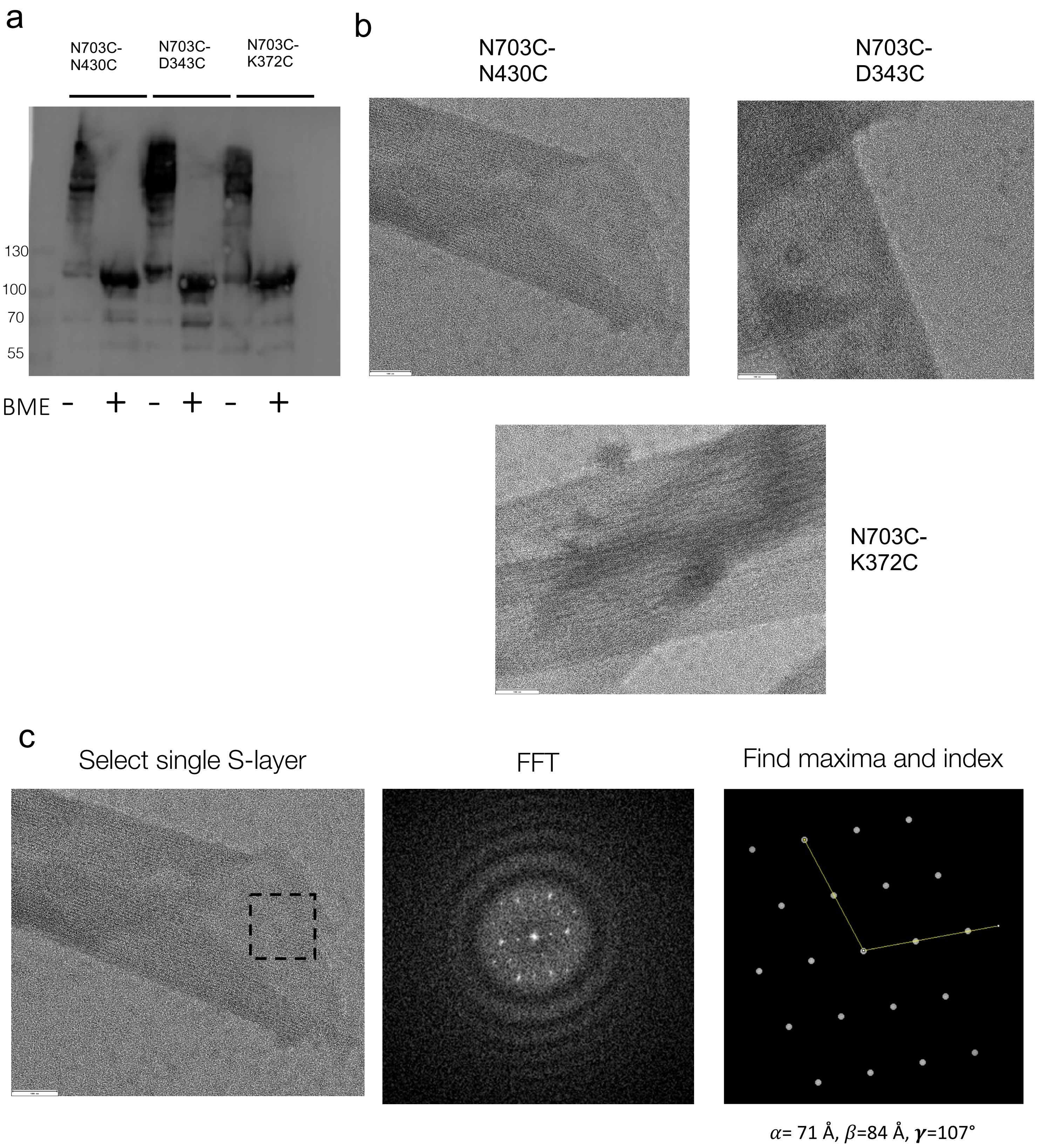

Supplementary figure 11. Validation of the D2 docking by cysteine mutants a. Western blot anti-His tag of the cysteine mutants in the presence (+) or absence (-) of β-mercaptoethanol. Ladder in kDa is indicated. **b**. Negative-staining TEM micrograph of *in vitro* reconstituted EA1 cysteine mutants. Scale bar is 100 nm. **c**. FFT and lattice parameters for the EA1 cysteine mutant N703C-N430C. The exact same values were obtained for N703C-D343C and N703C-K372C. Values for cysteine mutants are very similar to the WT measured using cryo-EM, the small observed difference could be due to the staining process. The experiments were repeated independently at least three times with similar results.

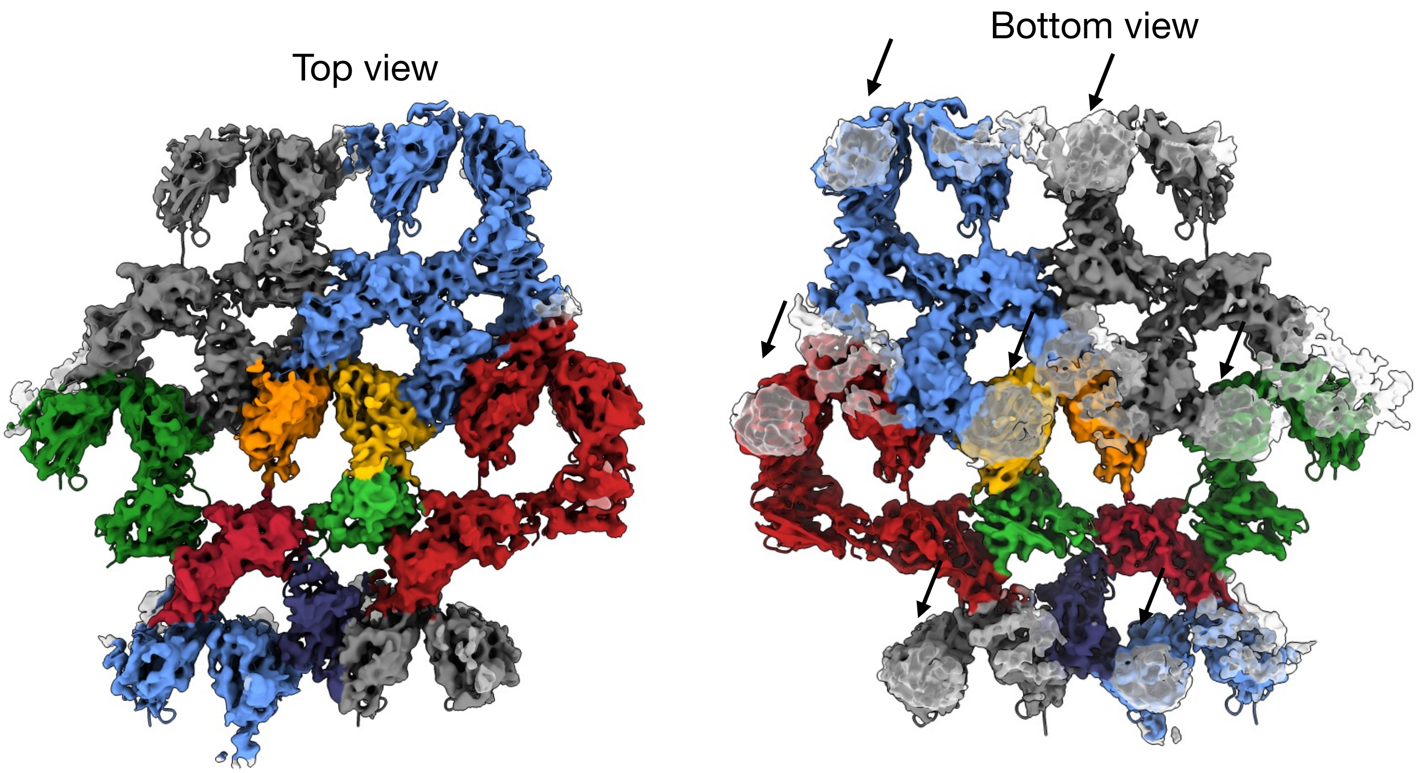

Supplementary figure 12. Fitting of the D2 to D6 into the Cryo-EM map. Reconstructed X-ray lattice containing D3 to D6 combined with manually docked D2 were fitted into the Cryo-EM map and colored by domain as indicated in the figure. Several non-colored extra densities are observed (arrows) pointing towards the cell that correspond to the D1 density.

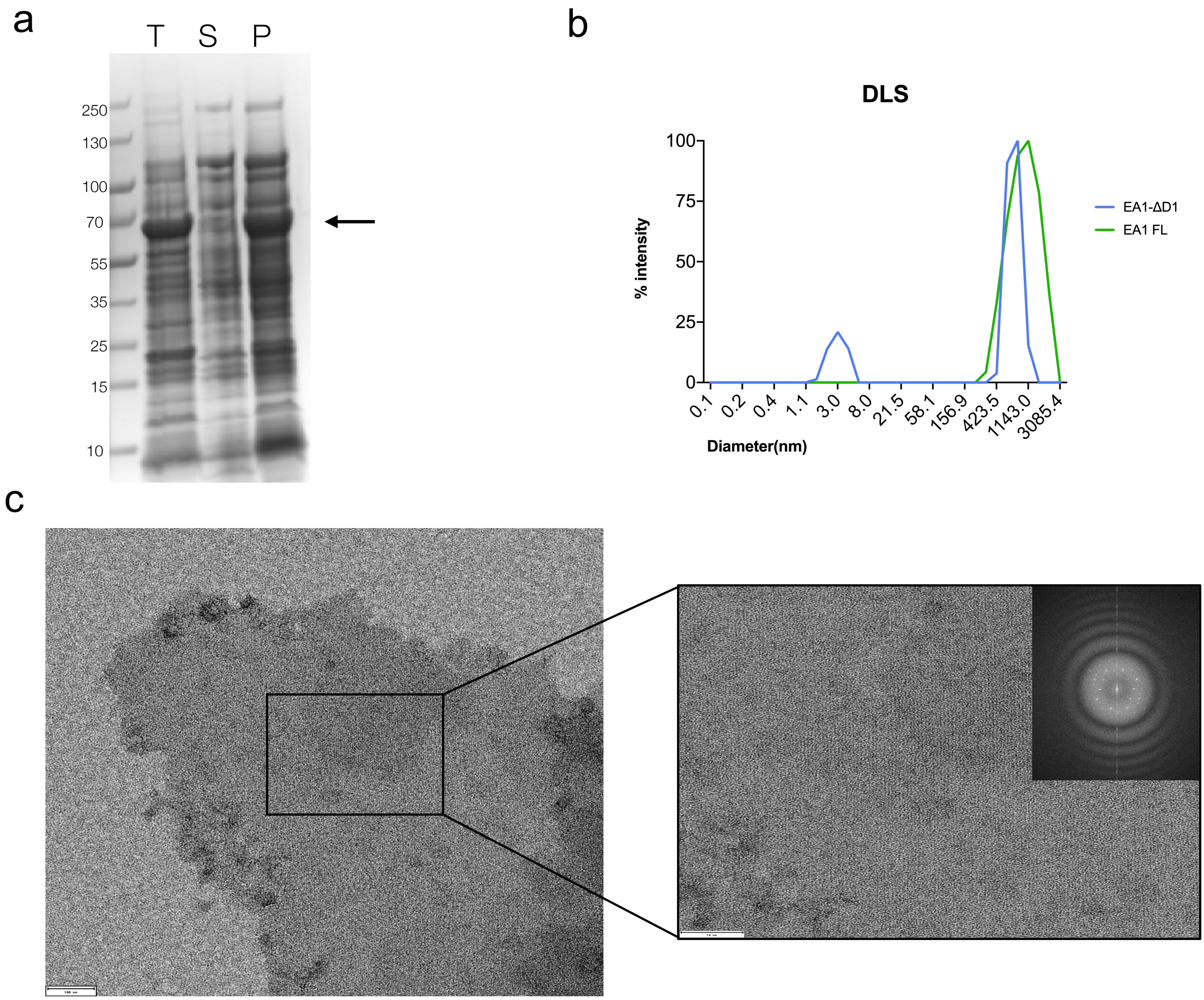

Supplementary figure 13. EA1$_{\Delta D1}$ forms S-layers. a. When purified, EA1$_{\Delta D1}$ (61.41 kDa) is found in the pellet (non-soluble fraction) as seen in the SDS-PAGE (arrow). T (Total lysed cells); S (soluble fraction); P (pellet – insoluble fraction). **b**. After purification of EA1$_{\Delta D1}$ under denaturing condition (urea) and refolding, EA1$_{\Delta D1}$ forms high diameter particles as measured using DLS. This is in the same range as the WT EA1$_{FL}$ indicating that EA1$_{\Delta D1}$ forms S-layer. **c**. To confirm the presence of S-layers and rule out aggregation, we imaged EA1$_{\Delta D1}$ after refolding and we could observe single-layered 2D crystals. Inset, power spectrum of the image presented in panel c. Scale bar 100 nm (right image) and 70 nm (left image). The experiments were repeated independently at least three times with similar results.

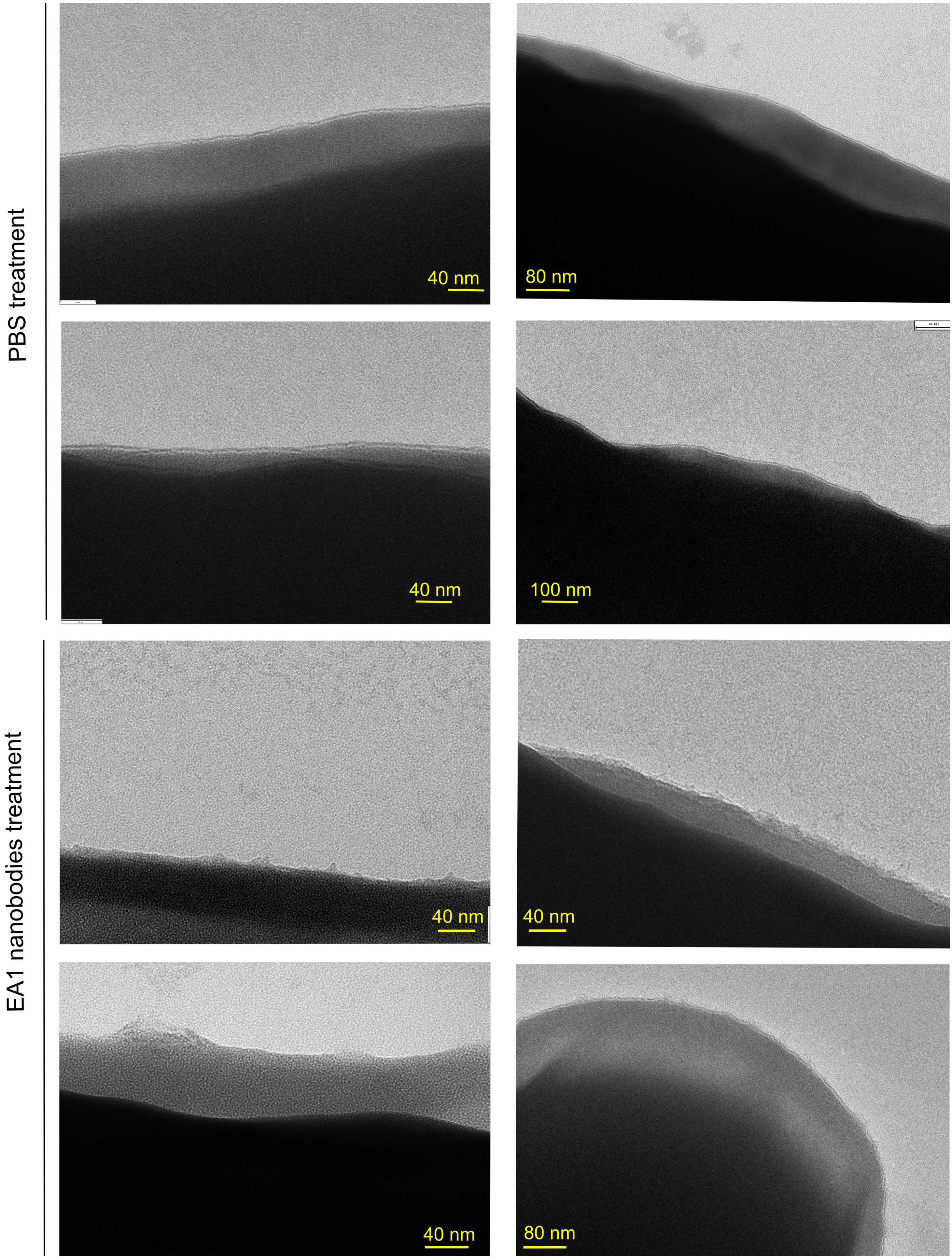

Supplementary Figure 14. Gallery of negative stain TEM images of B. anthracis 7702 cells treated with PBS buffer or 200 µM each of EA1 nanobodies Nb633, Nb632 and Nb643. PBS-treated cells show a continuous high-contrast monolayer on the bacterium's surface (S-layer) whereas stationary phase cells treated with nanobodies show an irregular surface indicating the loss of S-layer integrity.

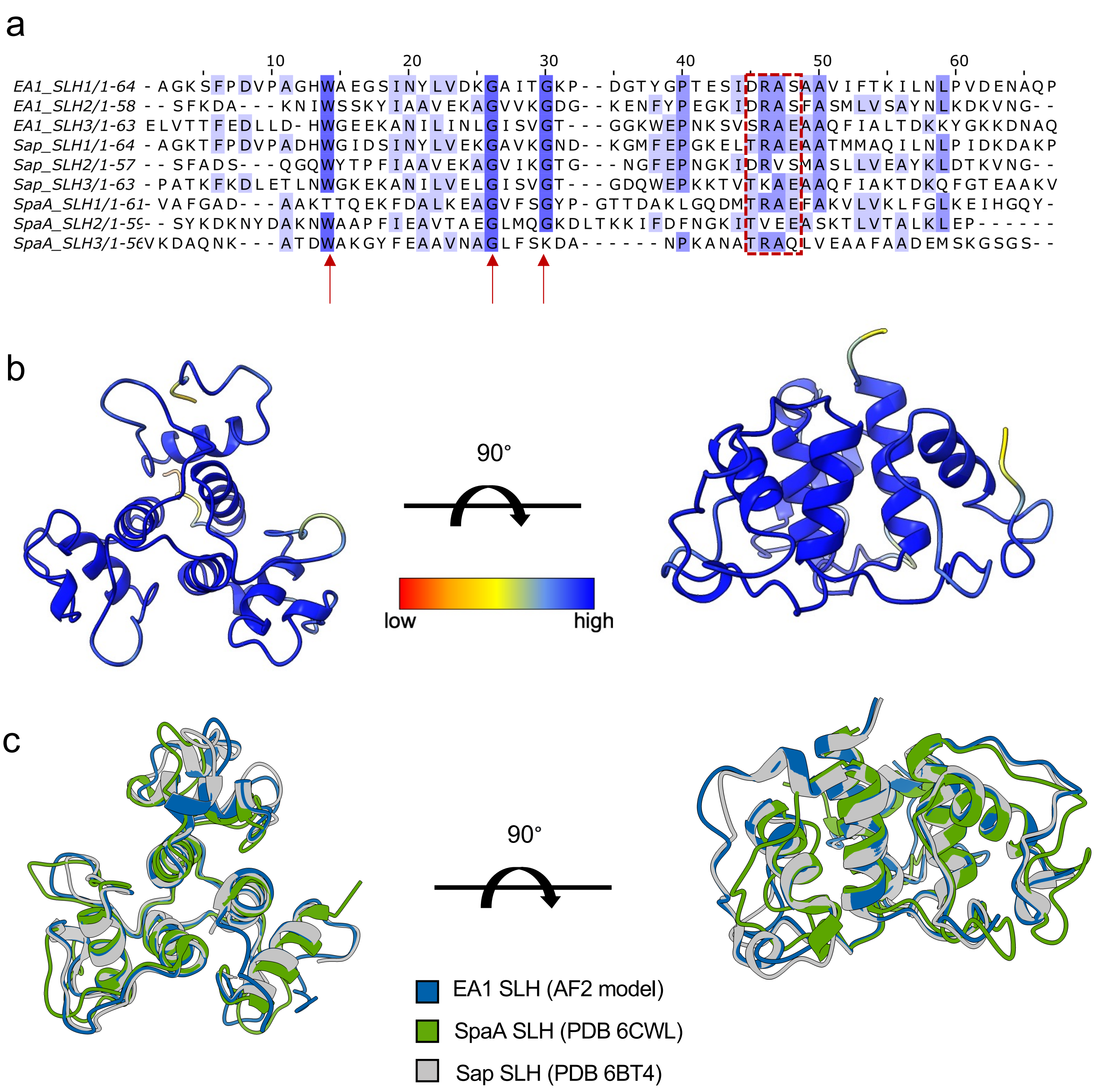

rmsd values table

	EA1 SLH	Sap SLH	SpaA SLH
EA1 SLH	0	1,02	2,98
Sap SLH		0	3,12
SpaA SLH			0

Supplementary Figure 15. Comparison of SLH domains. **a**. Sequence alignment of the three SLH domains from EA1, Sap (*B. anthracis*) and SpaA (*Paenibacillus alvei*). Key residues involved in SCWP binding are indicated with arrows and a box. **b**. AlphaFold2 prediction of the SLH domains of EA1 shown in ribbon representation and coloured by model confidence. The three SLH domains present a very high confidence prediction. **c**. (top) Structural superposition of the predicted $EA1_{SLH}$ domain with the experimentally solved Sap (pdb 6BT4) and SpaA (pdb 6CWL). (bottom) Table showing the rmsd values when comparing the SLH domain structure.

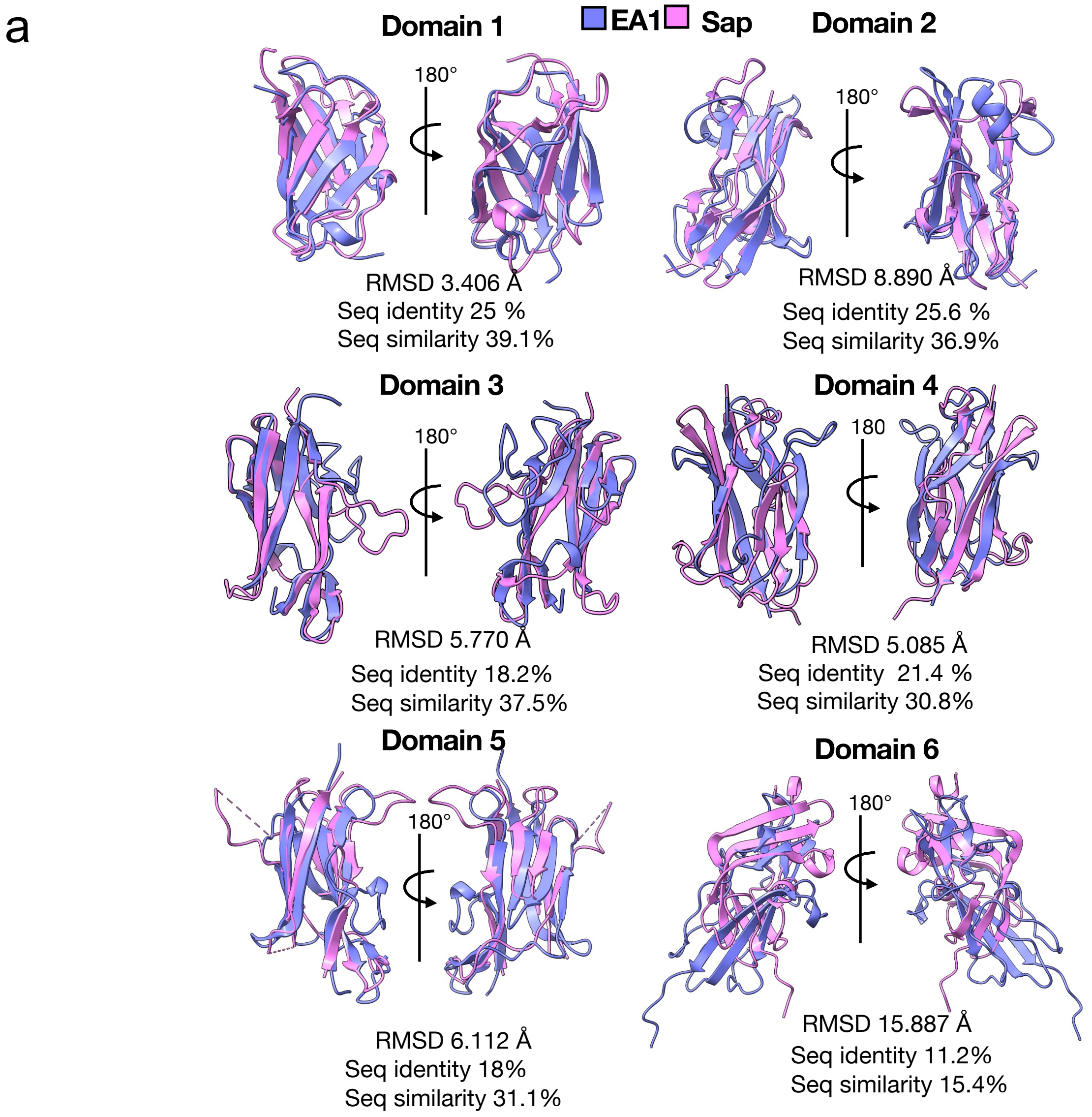

b

RMSD (Å)	D1 EA1	D2 EA1	D3 EA1	D4 EA1	D5 EA1	D6 EA1
D1 Sap	3.406	13.761	12.805	13.098	18.626	18.305
D2 Sap	12.816	8.890	16.341	13.375	11.301	11.237
D3 Sap	15.491	13.707	5.770	16.400	12.897	21.979
D4 Sap	14.569	11.632	9.805	5.085	11.043	21.776
D5 Sap	14.211	19.736	15.010	16.524	6.112	18.407
D6 Sap	18.625	21.420	12.374	13.772	16.739	15.887

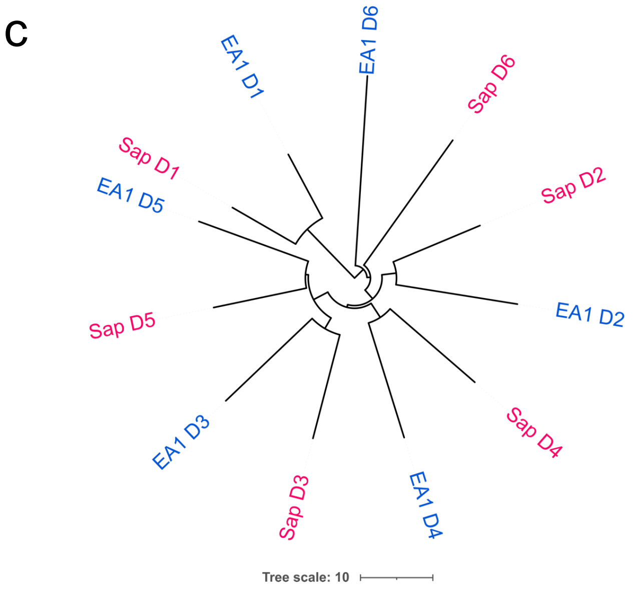

Supplementary Figure 16. Domain comparison of EA1 and Sap. A. Structural alignment of *B. anthracis* EA1 (purple) and Sap (pink) (PDB 6HHU) shown in ribbon representation. Sequence identity and similarity were calculated with EMBOSS needle and root mean square deviation (RMSD) measure with ChimeraX. **B**. Table comparing RMSD across all Sap and EA1 domains coloured according to structural similarity. **C**. Domains structural similarity dendrogram based on Dali Z-scores.

a

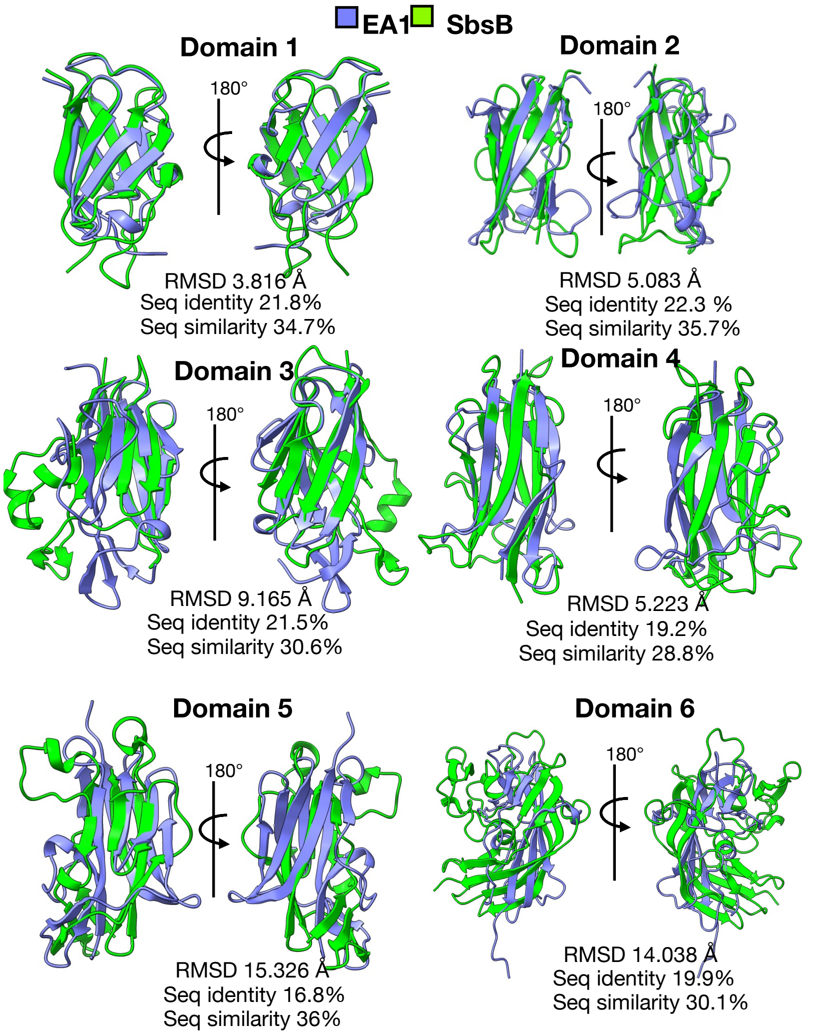

b

RMSD (Å)	D1 EA1	D2 EA1	D3 EA1	D4 EA1	D5 EA1	D6 EA1
D1 SbsB	3.816	10.47	16.495	16.570	15.929	16.075
D2 SbsB	17.436	5.083	5.837	9.618	11.802	10.690
D3 SbsB	13.918	17.412	9.165	16.877	6.275	15.734
D4 SbsB	17.774	13.938	17.187	5.223	18.547	20.781
D5 SbsB	15.087	10.011	14.432	14.280	15.326	15.326
D6 SbsB	17.832	19.370	17.760	21.115	20.135	14.038

c

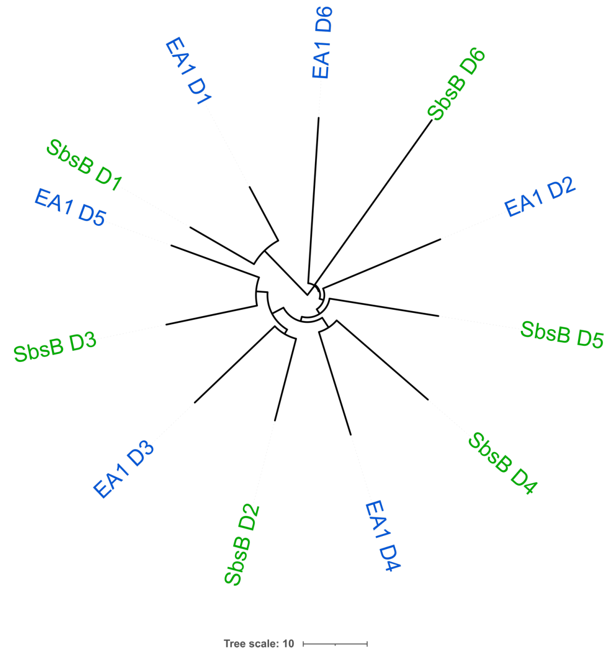

Supplementary figure 17. Domain comparison of EA1 and SbsB. a. Structural alignment of *B. anthracis* EA1 (blue) and SbsB (green) (PDB 4AQ1) shown in ribbon representation. Sequence identity and similarity were calculated with EMBOSS needle and root mean square deviation (RMSD) measure with ChimeraX. **b**. Table comparing RMSD across all Sap and SbsB domains colored according to structural similarity. **C**. Domains structural similarity dendrogram based on Dali Z-scores.

a

	Csg_D1	EA1_D6	EA1_D1	EA1_D4	EA1_D5	EA1_D3	EA1_D2	Csg_D6	Csg_D2	Csg_D3	Csg_D5	Csg_D4
Csg_D1	19,2	0,1	0,1	0,1	0,1	0,1	0,1	0,1	0,1	0,1	0,1	0,1
EA1_D6	0,1	29,1	0,1	3,4	2,3	2,9	2,4	2,4	2,8	2,3	2,4	0,1
EA1_D1	0,1	0,1	20,5	2,8	0,1	0,1	2,2	4,7	2,3	2,6	4,6	2
EA1_D4	0,1	3,4	2,8	24,2	6,3	5,3	3,6	5,8	4,8	5,5	4,6	4,7
EA1_D5	0,1	2,3	0,1	6,3	24	7,6	4,4	4,5	4,3	4,3	3,7	3,1
EA1_D3	0,1	2,9	0,1	5,3	7,6	25,7	4,1	6	5,7	3,3	3,9	3,3
EA1_D2	0,1	2,4	2,2	3,6	4,4	4,1	23,9	6,9	5,3	3,7	4,1	4,4
Csg_D6	0,1	2,4	4,7	5,8	4,5	6	6,9	23,6	9,1	7,8	8,8	6,9
Csg_D2	0,1	2,8	2,3	4,8	4,3	5,7	5,3	9,1	28,2	6,1	6,1	5,4
Csg_D3	0,1	2,3	2,6	5,5	4,3	3,3	3,7	7,8	6,1	32,2	7,2	7,1
Csg_D5	0,1	2,4	4,6	4,6	3,7	3,9	4,1	8,8	6,1	7,2	31,3	7,7
Csg_D4	0,1	0,1	2	4,7	3,1	3,3	4,4	6,9	5,4	7,1	7,7	29,4

b

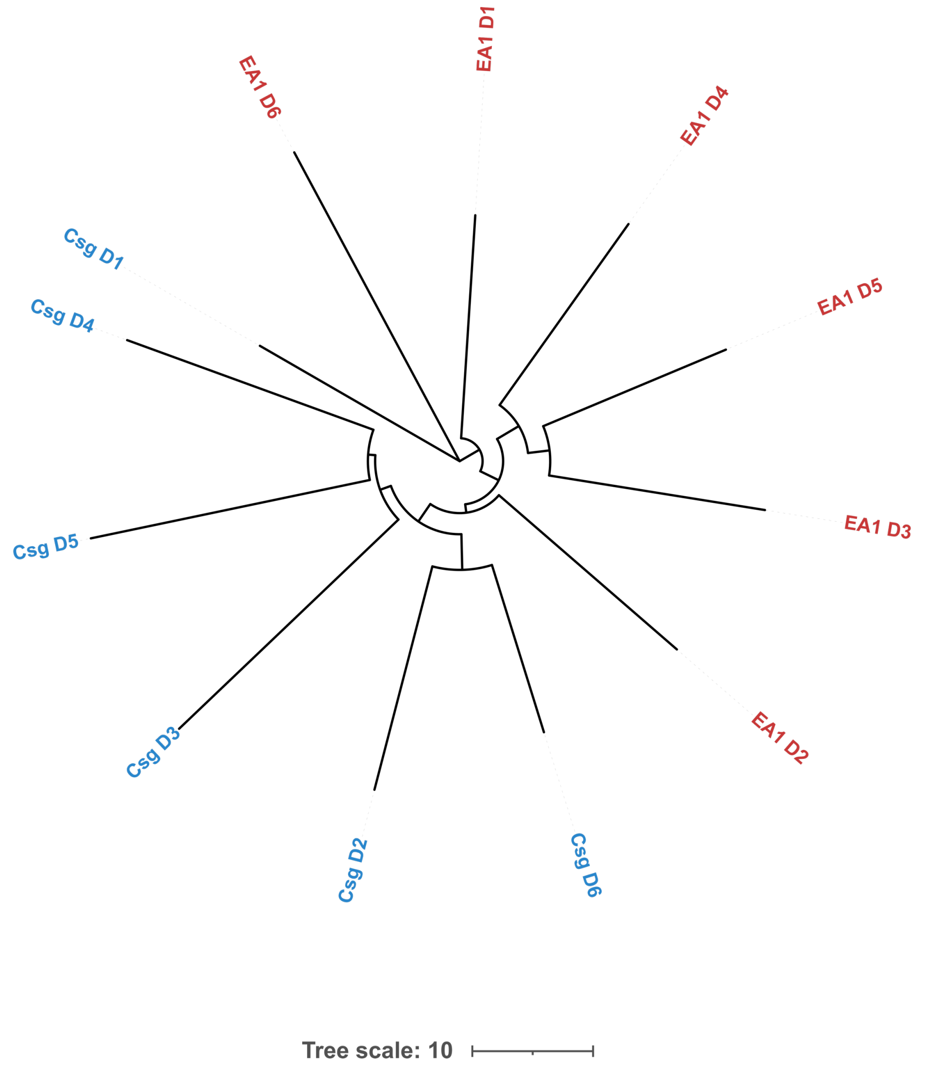

Supplementary Figure 18. Structural domain comparison of EA1 and the archaeal S-layer protein Csg (PDB 7PTR). **a**. Dali Z-score table. High values indicate structural similarity. **b**. Domains structural similarity dendrogram based on Dali Z-scores. Unlike when compared to Sap or SbsB, Ea1 domains cluster together which indicates that Csg and EA1 do not share a close common origin.

a

	HPI_D5	EA1_D6	HPI_D3	EA1_D1	EA1_D4	EA1_D5	EA1_D3	EA1_D2	HPI_D7	HPI_D4	HPI_D6	HPI_D2
HPI_D5	20,6	0,1	2,7	0,1	0,1	0,1	0,1	0,1	0,1	4,8	2,8	2,8
EA1_D6	0,1	29,1	0,1	0,1	3,4	2,3	2,9	2,4	0,1	2,3	2,5	2,6
HPI_D3	2,7	0,1	24,7	2,7	2,9	3,4	2,8	2,9	3,1	5,8	6	4,9
EA1_D1	0,1	0,1	2,7	20,5	2,4	0,1	2	2,2	2,3	0,1	2,5	4,3
EA1_D4	0,1	3,4	2,9	2,4	24,2	6,3	5,3	3,6	2,1	3,9	4	5,3
EA1_D5	0,1	2,3	3,4	0,1	6,3	24	7,6	4,5	2,7	3,2	3,6	5
EA1_D3	0,1	2,9	2,8	2	5,3	7,6	25,7	4,1	2,7	3,6	4,4	4,7
EA1_D2	0,1	2,4	2,9	2,2	3,6	4,5	4,1	23,9	3,9	4,9	5,2	5,4
HPI_D7	0,1	0,1	3,1	2,3	2,1	2,7	2,7	3,9	31,1	7,3	6,7	6,3
HPI_D4	4,8	2,3	5,8	0,1	3,9	3,2	3,6	4,9	7,3	31,5	6,8	5,5
HPI_D6	2,8	2,5	6	2,5	4	3,6	4,4	5,2	6,7	6,8	25,4	9,1
HPI_D2	2,8	2,6	4,9	4,3	5,3	5	4,7	5,4	6,3	5,5	9,1	21,4

b

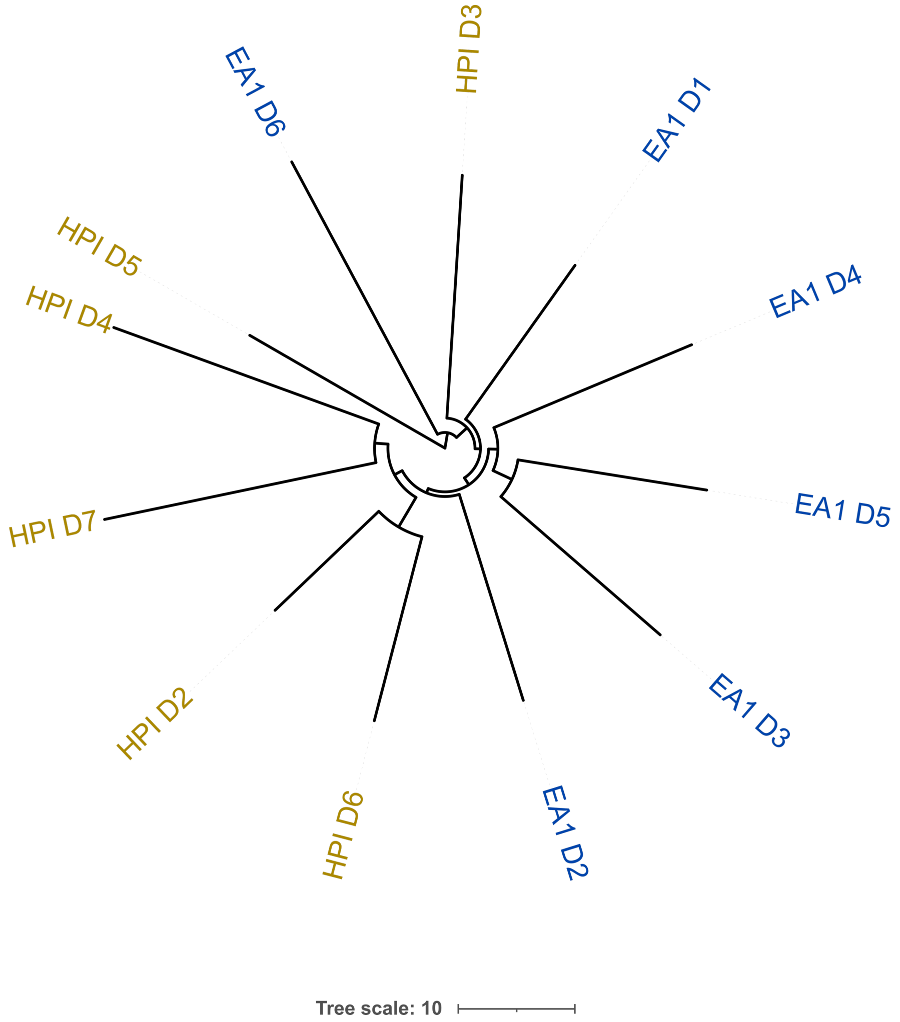

Supplementary Figure 19. Structural domain comparison of EA1 and the diderm bacterial S-layer protein HPI domains (PDB 8CKA). **a**. Dali Z-score table. High values indicate structural similarity. **b**. Domains structural similarity dendrogram based on Dali Z-scores. Unlike Sap or SbsB, EA1 domains cluster together, indicating that HPI and EA1 do not share a close common origin.

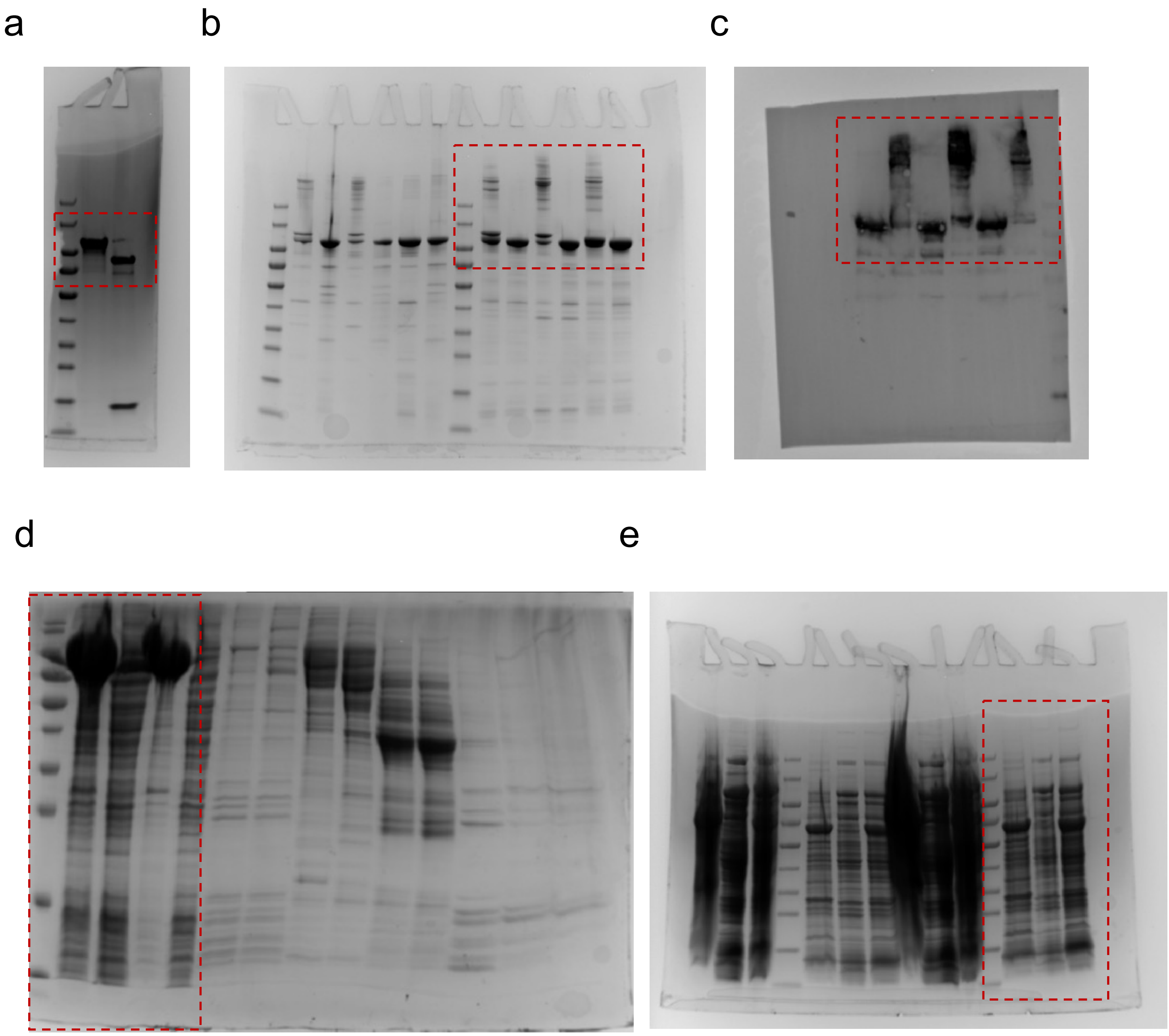

Supplementary Figure 20. Uncropped gels and blots. a. Figure 1.b Inset. **b**. Figure 4.f. **c**. Supplementary Figure 11.a. **d**. Supplementary Figure 2.b. **e**. Supplementary Figure 13.a.

	$EA1^{AD}$+Nb^{632}+Nb^{643}
**Data collection**	
Space group	P1
Cell dimensions	
a, b, c (Å)	72.93, 74.30, 87.65
α, β, γ (°)	107.85, 101,14, 112,42
Resolution (Å)*	62.35 - 1.811 (1.876 - 1.811)
R_{sym}	0.010 (0.1094)
R_{merge}	0.062 (0.550)
I/σ(I)	25.7 (1.8)
Completeness (%)	99 (64.6)
Spherical<br>Elliptical*	53.1 (8.9)<br>91.7 (64.6)
Multiplicity	3.1 (2.9)
**Refinement**	
Resolution (Å)	1.811
Number of unique reflections	73143 (3659)
R-work/R-free	0.1990 / 0.2371
Number of atoms	
protein	6670
ligands/ions	58
water	860
B-factors ($Å^2$)	
protein	56.34
ligands/ions	69.16
water	44.74
RMS deviations	
Bond length (Å)	0.012
Bond angles (°)	1.54
**PDB code**	**8OPR**

Supplementary Table 1. Crystallographic data.

Values in parenthesis refer to the highest recorded resolution shell.

* Elliptical diffraction limits and principal reciprocal axes of the fitted ellipsoid are 2.862 Å along direction 1 (0.815 a* + 0.566 b* - 0.125 c*), 1.992 Å along direction 2 (-0.559 a* + 0.759 b* - 0.335 c*) and 1.811 along direction 3 (-0.336 a* + 0.031 b* + 0.941 c*)

**Clonning of EA1 FL** (A93)		
**321**	F_open_pASK_3	TAATAAGCTTGACCTGTGAAG
**322**	R_open_pASK_3	CATTTGTATATCTCCTTCTTAAAG
**368**	F-pASK-His-EA1	GGAGATATACAAATGCATCACCATCATCATCACAAATCATTCCCAGACGTTCCA
**369**	R-pASK-EA1	CAGGTCAAGCTTAttaTTATAGATTTGGGTTATTAAGAACGTTCAC
**370**	EA1_seq1	AAAGTTAACGGCGAGTTAGTTACG
**371**	EA1_seq2	TGATGGTGTTGTAAACTATGGCAG
**372**	EA1_seq3	TCGTAACAACTGACCAATATGGC
**305**	F_seq_pASK	GAGTTATTTTACCACTCCCT
**306**	R_seq_pASK	CGCAGTAGCGGTAAACG
**Clonning of the EA1$_{FL}$ mutant N614W -A484Q (A129)**		
**456**	F-EA1_N614W	GGTTGGGTTGCATTTAAAAACTTTGAAC
**457**	R-EA1_N614W	AACCCAACCCTCTGTTACGTTC
**458**	F-EA1_A484Q	GAGCAGGCTGGTGAAGCTACACT
**459**	R-EA1_A484Q	CCAGCCTGCTCAGCAGTAATGTAGTTA
**Clonning of Cys mutants (A126, A127, A128)**		
**462**	F-EA1-N703C	ACGAAATGTGGTGCAACTGCTGGTAAAG
**463**	R-EA1-N703C	GCACCACATTTCGTTAAGTGGATATCAACT
**464**	F-EA1-N340C	GCGTGTAACTTAGACGGTTCACCA
**465**	R-EA1-N340C	GTTACACGCTACAAATTTGACGTCATGGT
**466**	F-EA1-D343C	TATGTGGTTCACCAGCAAACATCTTTG
**467**	R-EA1-D343C	AACCACATAAGTTATTCGCTACAAATTTGAC
**468**	F-EA1-K372C	CTACTGTGTAGAAGTACAAGTTACAAAAC
**469**	R-EA1-K372C	TACACAGTAGTCACCCTGCTTAAT
**Clonning EA1ΔD6 and EA1ΔD1 (A113 and A120)**		
**368**	F-pASK-His-EA1	GGAGATATACAAATGCATCACCATCATCATCACAAATCATTCCCAGACGTTCCA
**453**	R_EA1_D5	CAGGTCAAGCTTAttaTGTCTCTTGAACGATTTCGACTG
**446**	F_EA1_D2	GGAGATATACAAATGCATCACCATCATCATCACGTGAAAAAATTAGCAGTAGAAAAACTT
**455**	R_EA1_D6	CAGGTCAAGCTTAttaTTATAGATTTGGGTTATTAAGAACGTT

Name	Vector family	Inducer	Resistance	Overexpressing gene	Reference
**pAFSLP10**	pET300	IPTG	Ampi	EA1-AD	Fioravanti et al, 2019
**A93**	pASK-IBA3plus	anhydrotetracycline	Ampi	EA1-FL	This study
**A113**	pASK-IBA3plus	anhydrotetracycline	Ampi	EA1ΔD6	This study
**A120**	pASK-IBA3plus	anhydrotetracycline	Ampi	EA1ΔD1	This study
**A126**	pASK-IBA3plus	anhydrotetracycline	Ampi	EA1-FL (N703C/N340C)	This study
**A127**	pASK-IBA3plus	anhydrotetracycline	Ampi	EA1-FL (N703C/D343)	This study
**A128**	pASK-IBA3plus	anhydrotetracycline	Ampi	EA1-FL (N703C/K372C)	This study
**A129**	pASK-IBA3plus	anhydrotetracycline	Ampi	EA1-FL (N614W-A484Q)	This study

Supplementary Table 2. Primers (top) and plasmids (bottom) used in this study
