# Supplementary material for: Structure and function of the EA1 surface layer of Bacillus anthracis
Source: Nat Commun. 2023 Nov 3;14:7051. doi: 10.1038/s41467-023-42826-x (PMC10624894; doi:10.1038/s41467-023-42826-x)
Supplement: Supplementary file 3 — Reporting Summary [file 41467_2023_42826_MOESM3_ESM.pdf]

nature portfolio

Corresponding author(s): Han Remaut; Adrià Sogues

Last updated by author(s): Oct 12, 2023

Reporting Summary

Nature Portfolio wishes to improve the reproducibility of the work that we publish. This form provides structure for consistency and transparency in reporting. For further information on Nature Portfolio policies, see our Editorial Policies and the Editorial Policy Checklist.

Statistics

For all statistical analyses, confirm that the following items are present in the figure legend, table legend, main text, or Methods section.

n/a	Confirmed	
☐	☒	The exact sample size (*n*) for each experimental group/condition, given as a discrete number and unit of measurement
☐	☒	A statement on whether measurements were taken from distinct samples or whether the same sample was measured repeatedly
☐	☒	The statistical test(s) used AND whether they are one- or two-sided<br>*Only common tests should be described solely by name; describe more complex techniques in the Methods section.*
☒	☐	A description of all covariates tested
☐	☒	A description of any assumptions or corrections, such as tests of normality and adjustment for multiple comparisons
☐	☒	A full description of the statistical parameters including central tendency (e.g. means) or other basic estimates (e.g. regression coefficient) AND variation (e.g. standard deviation) or associated estimates of uncertainty (e.g. confidence intervals)
☐	☒	For null hypothesis testing, the test statistic (e.g. *F*, *t*, *r*) with confidence intervals, effect sizes, degrees of freedom and *P* value noted<br>*Give P values as exact values whenever suitable.*
☒	☐	For Bayesian analysis, information on the choice of priors and Markov chain Monte Carlo settings
☒	☐	For hierarchical and complex designs, identification of the appropriate level for tests and full reporting of outcomes
☒	☐	Estimates of effect sizes (e.g. Cohen's *d*, Pearson's *r*), indicating how they were calculated

Our web collection on statistics for biologists contains articles on many of the points above.

Software and code

Policy information about availability of computer code

Data collection	Dynapro Dynamics (version 7.1.9.3); SerialEM 3.8 ; GelDoc Go (Bio-Rad)
Data analysis	Dynapro Dynamics (version 7.1.9.3); GraphPad Prism 8; AutoProc; Staraniso; AlphaFold2; Buster 2.10.4; Image Lab 6.1 (Bio-Rad); UCSF ChimeraX 0.92; PHENIX 1.19.2; Coot 0.9.5; cryoSPARC; 3DFSC server; R Studio 2023.03.0

For manuscripts utilizing custom algorithms or software that are central to the research but not yet described in published literature, software must be made available to editors and reviewers. We strongly encourage code deposition in a community repository (e.g. GitHub). See the Nature Portfolio guidelines for submitting code & software for further information.

Data

Policy information about availability of data

All manuscripts must include a data availability statement. This statement should provide the following information, where applicable:

- Accession codes, unique identifiers, or web links for publicly available datasets
- A description of any restrictions on data availability
- For clinical datasets or third party data, please ensure that the statement adheres to our policy

The atomic coordinates have been deposited in the Protein Data Bank (PDB) under accession code 8OPR [https://doi.org/10.2210/pdb8OPR/pdb] (crystal structure of EA1AD in complex with nanobodies 632 and 643). The model for the EA1 S-layer lattice is available through Mendeley at [https://data.mendeley.com/datasets/wf93wvy2vz/1] DOI: 10.17632/wf93wvy2vz.1. Source data for all relevant Figures are provided with

this paper. Additional data and materials are available from the corresponding authors upon reasonable request. Requests for Bacillus anthracis strains should be addressed to the primary source, as cited in the manuscript.

Research involving human participants, their data, or biological material

Policy information about studies with human participants or human data. See also policy information about sex, gender (identity/presentation), and sexual orientation and race, ethnicity and racism.

Reporting on sex and gender	NA
Reporting on race, ethnicity, or other socially relevant groupings	NA
Population characteristics	NA
Recruitment	NA
Ethics oversight	NA

Note that full information on the approval of the study protocol must also be provided in the manuscript.

Field-specific reporting

Please select the one below that is the best fit for your research. If you are not sure, read the appropriate sections before making your selection.

☒ Life sciences ☐ Behavioural & social sciences ☐ Ecological, evolutionary & environmental sciences

For a reference copy of the document with all sections, see nature.com/documents/nr-reporting-summary-flat.pdf

Life sciences study design

All studies must disclose on these points even when the disclosure is negative.

Sample size	No sample size calculation was performed. Where null hypotheses are tested, a sample size of at least ten has been used. Where biochemical dose response effects, or bacterial growth are reported, these are reported as means ±SD of at least three biological (i.e. independent) replicates, as is standard in the field. Sample sizes for each experiment are described in the legend of the respective figures.
Data exclusions	No data were excluded.
Replication	For cellular experiments, at least three independent replicates (i.e. independent innocula starting from individual colonies) were performed and used in the data analysis and presenation.
Randomization	Samples were not randomized as this is not applicable to the experiments used.
Blinding	No blinding was applied. All data from individual samples represent digitized quantitative instrument-acquired measures, avoiding any subjective bias.

Reporting for specific materials, systems and methods

We require information from authors about some types of materials, experimental systems and methods used in many studies. Here, indicate whether each material, system or method listed is relevant to your study. If you are not sure if a list item applies to your research, read the appropriate section before selecting a response.

Materials & experimental systems

n/a	Involved in the study	
☐	☒	Antibodies
☒	☐	Eukaryotic cell lines
☒	☐	Palaeontology and archaeology
☒	☐	Animals and other organisms
☒	☐	Clinical data
☒	☐	Dual use research of concern
☒	☐	Plants

Methods

n/a	Involved in the study	
☒	☐	ChIP-seq
☒	☐	Flow cytometry
☒	☐	MRI-based neuroimaging

Antibodies

Antibodies used	- Antibodies are monoclonal llama VHH fragments, i.e. Nanobodies, identified and reported in this study.<br>Antibodies used for Western Blotting: Mouse anit-His:AP conjugate (BioRad MCA1396A), mentioned in the respective Methods section.
Validation	Commercial antibodies are validated by the vendor; VHH identified and used in this study were validated by structural biology and BLI-based affinity measurement.
